# Aggressive Prostate Cancer After a 14-Year Gonadotropin Therapy: A Case Report

**DOI:** 10.7759/cureus.62672

**Published:** 2024-06-19

**Authors:** Fumie Yoshioka, Hiroshi Kiuchi, Testuji Soda, Akira Tsujimura, Kenichiro Sekii

**Affiliations:** 1 Department of Urology, Osaka Central Hospital, Osaka, JPN; 2 Department of Urology, Juntendo University Urayasu Hospital, Urayasu, JPN

**Keywords:** high-grade cancer, psa monitoring, advanced cancer, prostate cancer, gonadotropin therapy

## Abstract

A 40-year-old man with a four-year history of infertility was referred to our department. The semen analysis revealed low motility, and the blood test showed low luteinizing hormone levels. Gonadotropin therapy was initiated upon the diagnosis of hypogonadotropic hypogonadism. During treatment, serum prostate-specific antigen (PSA) was consistently low (1.4-1.9 ng/mL). Fourteen years after the start of treatment, at 54 years old, PSA was abruptly elevated (3.5 ng/mL), and gonadotropin therapy was discontinued due to possible prostate cancer. After cessation, PSA decreased temporarily but then gradually increased to 7.6 ng/mL, but the patient requested PSA follow-up. Twenty years after discontinuation of gonadotropin therapy, PSA increased sharply to 65.9 ng/mL. A prostate biopsy revealed adenocarcinoma with a Gleason score of 4+5. A bone scan showed multiple bone metastases, leading to an advanced prostate cancer (cT4N0M1b) diagnosis. Six months after androgen deprivation therapy, PSA increased again. Under castration-resistant prostate cancer diagnosis, enzalutamide and radium-223 chloride were administered. After treatment, bone metastases were significantly reduced, and PSA decreased. Although gonadotropin and testosterone replacement therapy may not increase prostate cancer risk, patients with low testosterone levels may develop high-grade advanced prostate cancer. Therefore, PSA should be monitored regularly; if PSA levels are continuously elevated, even below 4 ng/mL, a close examination of cancer may be warranted.

## Introduction

Gonadotropin therapy has shown promising efficacy in infertility patients with idiopathic oligozoospermia or nonobstructive azoospermia, making it a practical option [1]. Gonadotropin therapy simultaneously stimulates testosterone production, which may increase the likelihood of prostate cancer in infertile patients aged over 40 years. This concern is similar to that of the possible increased risk of prostate cancer in older men with symptomatic late-onset hypogonadism (LOH) syndrome receiving testosterone replacement therapy. Guidelines for men with LOH and total testosterone (TT) supplementation recommend regular prostate-specific antigen (PSA) measurements for vigilance against prostate cancer [2]. To date, there have been no reports of patients developing prostate cancer after gonadotropin therapy. Herein, we report the case of a 40-year-old man who developed metastatic prostate cancer after 14 years of gonadotropin replacement therapy.

## Case presentation

A 40-year-old man with a four-year history of infertility was referred to our clinic. The patient couple had previously achieved a pregnancy through artificial insemination; however, the pregnancy resulted in a miscarriage. The semen analysis revealed asthenozoospermia with 18% progressive motility (normal, >32%), a semen volume of 2.0 mL, a sperm concentration of 55 × 10^6^/mL, and 20% malformations. The blood tests showed TT and luteinizing hormone (LH) levels at the lower limit of normal (TT: 3.1 ng/mL, LH: 1.5 mIU/mL; normal range TT: 2.9-10.7 ng/mL, LH: 1.5-7.0 mIU/mL), whereas levels of other hormones, including follicle-stimulating hormone (FSH), prolactin, and estradiol, were within the normal range. The urological examination showed a normal androgenized male (G5 and PH5 at the Tanner stage) with normal testicular volumes (Rt 12 mL, Lt 11 mL). The chromosomal examination showed 46XY, and no varicocele was noted. After examination, the patient was diagnosed with asthenozoospermia due to the idiopathic impairment of LH levels. The patient started self-injecting 2000 units of human chorionic gonadotropin (hCG) and 150 units of human menopausal gonadotropin (hMG). Thereafter, sperm motility increased up to 30%-50%. During hCG/hMG therapy, several in vitro fertilizations were performed but were unsuccessful.

**Figure 1:**
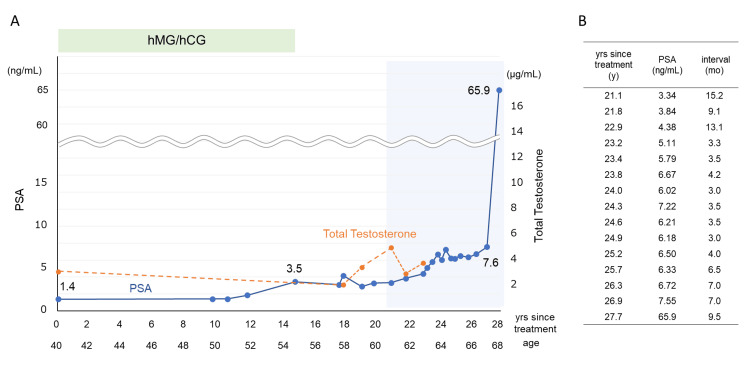
(A) The 28-year clinical course from the first visit until prostate cancer diagnosis. (B) PSA values and their measurement intervals from 20 years after the start of treatment to the diagnosis of prostate cancer. Gonadotropin therapy for infertility was discontinued after 14 years when PSA abruptly increased to 3.5 ng/mL (A). Since PSA increased gradually after 20 years of treatment, PSA was measured approximately every six months. PSA gradually increased to 7.6 ng/mL over 12 years and then rose rapidly to 65.9 ng/mL after 9.5 months from the previous PSA measurement (A, light blue box, B). hMG: human menopausal gonadotropin; hCG: human chorionic gonadotropin; PSA: prostate-specific antigen.

At baseline, serum PSA was 1.40 ng/mL. During gonadotropin therapy, PSA was measured on a regular basis, with constant low levels of PSA ranging from 1.40 to 1.87 ng/mL (Figure 1A). Fourteen years after gonadotropin treatment, at 54 years of age, PSA abruptly increased to 3.46 ng/mL, and gonadotropin therapy was discontinued for possible prostate cancer. After withdrawal of gonadotropin therapy, PSA decreased once to 2.88 ng/mL but then gradually increased (average increase of 0.59 ng/mL per year) to 7.6 ng/mL at 12 years after discontinuation of the gonadotropin therapy. After PSA exceeded 4.0 ng/mL, an MRI for a close examination of the prostate and a prostate biopsy, if necessary, were repeatedly suggested, but the patient requested PSA follow-up. After 9.5 months, it rose rapidly to 65.9 ng/mL (Figure 1A, 1B), indicating a high probability of prostate cancer.

**Figure 2:**
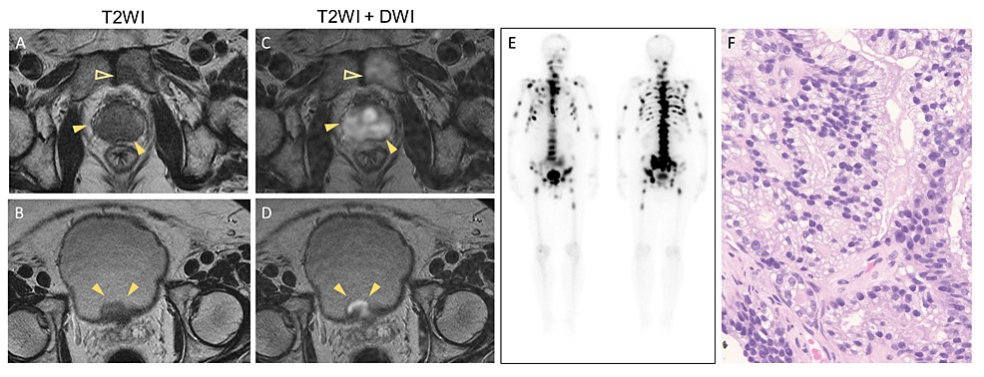
MRI, bone scan, and prostate biopsy specimens. A T2-weighted image (T2WI) demonstrates diffuse low intensity within the prostate (A, arrowheads) with metastasis of pubic bone (A, open arrowhead) and suspected bladder invasion (B, arrowhead); the merged T2WI and diffusion-weighted image (DWI) show high-intensity signals in identical regions (C, D). The whole-body bone scan reveals multiple bone metastases in the skull, bilateral proximal humerus and femur, left knee joint, spine, ribs, and pelvis (E). Hematoxylin and eosin staining shows high-grade adenocarcinoma of the prostate with a Gleason score of 4+5 (F).

**Figure 3:**
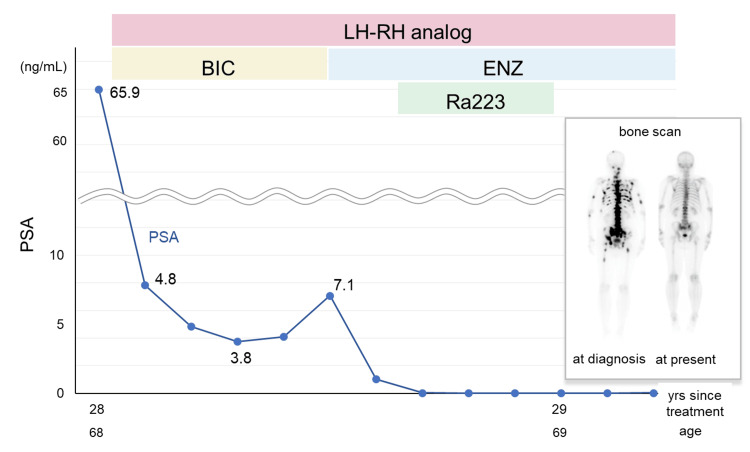
Clinical course after the initiation of prostate cancer therapy. The bone scans at diagnosis show multiple bone metastases, but those metastases improve after the administration of enzalutamide and radium-223 chloride. LH-RH: luteinizing hormone-releasing hormone; BIC: bicalutamide; ENZ: enzalutamide; Ra223: radium-223 chloride.

The patient agreed to an MRI, which revealed diffuse low signals of the prostate and suspected bladder or seminal vesicle invasion with multi-metastasis of the pubic or pelvic bone (Figure 2A-D). A bone scan revealed multiple bone metastases in the skull, bilateral proximal humerus and femur, left knee joint, spine, ribs, and pelvis (Figure 2E). The prostate biopsy revealed adenocarcinoma with a Gleason score of 4+5 in all the specimens (Figure 2F). The patient was diagnosed with advanced prostate cancer (cT4N0M1b), and a combined androgen blockade therapy (bicalutamide and degarelix acetate) was initiated. PSA decreased rapidly, reaching 3.75 ng/mL after six months of treatment (Figure 3). However, PSA was elevated again to 7.08 ng/mL; therefore, treatment was changed to enzalutamide with a diagnosis of castration-resistant prostate cancer. Because metastasis was found only in the bone, radium chloride (223Ra) was administered. Subsequently, bone metastasis greatly diminished, and the PSA level decreased to 0.03 ng/mL. Currently, treatment is continued, and metastasis remains reduced without any increase in PSA levels.

## Discussion

Due to the limited availability of evidence-based alternatives, gonadotropin therapy is widely utilized for infertile male patients with severe oligozoospermia or nonobstructive azoospermia characterized by low LH and/or TT levels. A recent review of gonadotropin therapy demonstrated its positive effects on semen quality and pregnancy rates in these patients [1]. At the same time, gonadotropin therapy also leads to increased TT levels, which may increase the risk of developing prostate cancer, as testosterone replacement therapy (TRT) for patients with LOH syndrome raises concerns. Given the patient’s age and duration of infertility treatment, the risk of prostate cancer is generally minimal. To date, there have been no reports of gonadotropin therapy increasing the risk of prostate cancer. Considering the current trend toward late marriage, the risk of prostate cancer may have to be considered in these infertile patients.

The association between TT supplementation and the development of prostate cancer has long been the focus of debate, and researchers have vigorously investigated this association in basic and clinical studies. The abundance of evidence indicates that TRT does not increase the risk of prostate cancer. In a recent randomized controlled trial, participants with testosterone levels <3.0 ng/mL were randomized to receive testosterone enanthate or placebo for six months [3]. TRT increased serum testosterone levels to the mid-normal range; however, prostate tissue testosterone and dihydrotestosterone levels did not change significantly, and no treatment-related changes were observed in prostate histology, tissue biomarkers, or cancer incidence. A meta-analysis of prospective clinical trials suggested that TRT did not affect the risk of developing prostate cancer [4]. However, few of these studies were well-designed with large-scale and long-term follow-ups, and the guidelines report insufficient evidence of an association between TT supplementation and prostate cancer. Large, long-term, and well-designed studies are needed to confirm the absence of this association.

With regard to serum testosterone levels and prostate cancer, there have been reports that low testosterone levels are not associated with the risk of developing prostate cancer but are associated with high malignant potential in prostate cancer [5-8]. Individuals with local prostate cancer and low TT (TT <2.5 ng/mL) had a significantly high Gleason score of ≥8 tumors [6]. In patients who underwent radical prostatectomy for localized prostate cancer, low TT levels were associated with non-organ-confined prostate cancer (pT3 or T4, N1) [7]. In addition, patients with metastatic prostate cancer and low serum testosterone levels had shorter survival than those with normal testosterone levels [8]. The reasons for the association between low testosterone levels and aggressive prostate cancer are not well understood; however, it has been suggested that the selection of poorly differentiated prostate cancer cells may occur in the low testosterone state. This hypothesis was derived from the Prostate Cancer Prevention Trial study, in which the 5α-reductase type 2 inhibitor decreased the overall incidence of prostate cancer but increased the incidence of high-grade cancer [9]. This hypothesis was confirmed in animal experiments conducted by Banach-Petrosky et al. [10]. They discovered that the inhibition of 5α-reductase in a genetically engineered mouse model of human prostate cancer, which lowered intraprostatic levels of dihydrotestosterone, induced the development of poorly differentiated prostate cancer.

The usefulness of PSA velocity during testosterone administration has been reported for the early detection of prostate cancer. In men with a baseline PSA level of less than 4 ng/mL, long-term follow-up indicated that a PSA velocity of 0.2 ng/mL per year had a sensitivity of 52.4% and a specificity of 79.4% in predicting prostate cancer [11]. Bhasin et al. proposed that for testosterone treatment periods of less than six months, an increase in PSA >0.4 ng/mL requires more intensive future surveillance for prostate cancer. Additionally, for testosterone administration periods of longer than six months, a PSA velocity of 0.4 ng/mL/year or greater is likely to indicate a higher-than-average risk of prostate cancer [12]. They emphasized that the use of these cutoff values requires at least two years of follow-up and multiple PSA measurements.

## Conclusions

Gonadotropin and testosterone replacement therapy may not increase the risk of prostate cancer; however, patients with low testosterone levels may develop prostate cancer with high malignant potential and advanced prostate cancer at the time of diagnosis. PSA levels are rarely elevated after an initial increase in TRT. Therefore, if a patient on TRT has a PSA level of 4.0 ng/mL or higher, or even if it is less than 4.0 ng/mL, but the PSA velocity exceeds 0.4 ng/mL/year, not only should testosterone be discontinued, but a close examination of the cancer, including a prostate biopsy, may be warranted.
